# Calcium dynamics and modulation in carrot somatic embryogenesis

**DOI:** 10.3389/fpls.2023.1150198

**Published:** 2023-03-31

**Authors:** Antonio Calabuig-Serna, Ricardo Mir, Paloma Arjona, Jose María Seguí-Simarro

**Affiliations:** Cell Biology Group - COMAV Institute, Universitat Politècnica de València, Valencia, Spain

**Keywords:** callose, DAUCUS carota, EGTA, FRET, *in vitro* culture, ionophore A23187, morphogenesis, W-7

## Abstract

Free calcium (Ca^2+^) is a pivotal player in different *in vivo* and *in vitro* morphogenic processes. In the induction of somatic embryogenesis, its role has been demonstrated in different species. In carrot, however, this role has been more controversial. In this work, we developed carrot lines expressing *cameleon* Ca^2+^ sensors. With them, Ca^2+^ levels and distribution in the different embryogenic structures formed during the induction and development of somatic embryos were analyzed by FRET. We also used different chemicals to modulate intracellular Ca^2+^ levels (CaCl_2_, ionophore A23187, EGTA), to inhibit calmodulin (W-7) and to inhibit callose synthesis (2-deoxy-D-glucose) at different times, principally during the first stages of embryo induction. Our results showed that high Ca^2+^ levels and the development of a callose layer are markers of cells induced to embryogenesis, which are the precursors of somatic embryos. Disorganized calli and embryogenic masses have different Ca^2+^ patterns associated to their embryogenic competence, with higher levels in embryogenic cells than in callus cells. The efficiency of somatic embryogenesis in carrot can be effectively modulated by allowing, within a range, more Ca^2+^ to enter the cell to act as a second messenger to trigger embryogenesis induction. Once induced, Ca^2+^-calmodulin signaling seems related with the transcriptional remodeling needed for embryo progression, and alterations of Ca^2+^ or calmodulin levels negatively affect the efficiency of the process.

## Introduction

Free calcium (Ca^2+^) has many different structural, metabolic and regulatory functions. One of the most important is its role as signaling molecule in both plant and animal cells (Permyakov and Kretsinger, 2009). Ca^2+^ is typically kept at very low concentrations in the cytoplasm (50-100 nM), being stored principally at the endoplasmic reticulum, nucleus, cell wall and vacuoles (Pirayesh et al., 2021). Thus, Ca^2+^ signaling relies on transient Ca^2+^ releases that change cytosolic Ca^2+^ concentrations in order to trigger regulation of gene expression and physiological responses (White and Broadley, 2003; Demidchik et al., 2018). Plants use Ca^2+^ to regulate and control, among others, the responses to biotic and abiotic stresses as well as different aspects of plant reproduction including pollen tube growth, double fertilization, embryo development and seed yield (Antoine et al., 2000; White and Broadley, 2003; Ge et al., 2007; Denninger et al., 2014; Tang and Luan, 2017; Tian et al., 2020). However, there is still limited information about Ca^2+^ dynamics during *in vivo* development of the plant zygotic embryo, probably due to the difficulties imposed by the surrounding maternal tissues. As alternatives, microspore-derived embryos (Rivas-Sendra et al., 2017; Rivas-Sendra et al., 2019) and principally somatic embryos have been used to study Ca^2+^ dynamics during early embryo development (Anil and Rao, 2000; Mahalakshmi et al., 2007). Somatic embryogenesis is an *in vitro* biotechnological process whereby plant somatic cells dedifferentiate from their original identity and start developing as embryos (Yang and Zhang, 2010). Somatic embryogenesis systems have been widely used to develop efficient micropropagation protocols as well as to study embryogenic development in several species including carrot (*Daucus carota*), where the first embryogenic suspension cultures were reported more than six decades ago (Reinert, 1958; Steward et al., 1958). Typically, the carrot somatic embryogenesis system is based on the generation of hypocotyl-derived calli, which are then disaggregated in auxin-containing medium into a suspension of cell clumps, from which embryogenic masses are formed. Upon hormone removal, embryos start developing from the embryogenic masses (De Vries et al., 1988). Carrot somatic embryos strongly resemble zygotic embryos with the exception of the suspensor (Halperin, 1964), which makes this system a useful model to study different aspects of plant embryogenesis, including Ca^2+^ dynamics. Besides, understanding the role of Ca^2+^ in the *in vitro* induction of embryogenesis may open new ways to improve the efficiency of this biotechnological tool of wide interest in applied plant breeding.

The role of Ca^2+^ in the induction of somatic embryogenesis has been demonstrated in species such as sandalwood (Anil and Rao, 2000), *Pinus patula* (Malabadi and Van Staden, 2006), coffee (Ramakrishna et al., 2011) or *Arabidopsis* (Calabuig-Serna et al., 2023). In carrot, however, this role has been somehow controversial. Some reports proposed that increased intracellular Ca^2+^ levels, induced through the addition of CaCl_2_ to the culture medium or by facilitating the diffusion of Ca^2+^ across the plasma membrane by adding ionophore A23187, could increase somatic embryogenesis in carrot (Jansen et al., 1990; Takeda et al., 2003), as shown in other species like coconut (Rivera-Solís et al., 2018). Instead, other studies showed that maintenance of Ca^2+^ homeostasis was essential to induce carrot somatic embryos, since increasing Ca^2+^ levels by adding CaCl_2_ or ionophore A23187 had no beneficial effect on embryo yield (Overvoorde and Grimes, 1994), concluding that a strict maintenance of Ca^2+^ homeostasis was a requisite for carrot somatic embryogenesis. In turn, addition of exogenous Ca^2+^ was found beneficial in other species such as *Hevea brasiliensis* to increase both the rate of induction of somatic embryos and to increase the capacity of regeneration and germination of the induced embryos (Etienne et al., 1997). In carrot, however, Ca^2+^ was suggested to allow for a more efficient progression of the embryos already induced, rather than for the induction of more embryos (Mizukami et al., 2008).

Most of these works were developed at least 20 years ago, using biochemical analyses and the fluorescence imaging technologies available at that time, including staining with Ca^2+^-binding fluorescent dyes such as chlorotetracycline (Timmers et al., 1989; Overvoorde and Grimes, 1994) or Fura2-AM (Anil and Rao, 2000; Ramakrishna et al., 2011). Although informative, some of these dyes have limited cell penetration and do not allow for *in vivo* Ca^2+^ observation. These limitations can now be overcome with genetically-encoded Ca^2+^ sensors such as *cameleon* probes (Miyawaki et al., 1997), based on fluorescence resonance energy transfer (FRET) for Ca^2+^ detection. *Cameleon* probes are produced by the tandem expression of a construct including a donor cyan fluorescent protein (CFP), a calmodulin (CaM) residue fused to the specific binding peptide M13, and an acceptor yellow fluorescent protein (YFP). Free Ca^2+^ binds CaM and induces a conformational change that favors the acceptor YPF excitation by the donor CPF emission (Miyawaki et al., 1997). *Cameleons* are also targetable to specific intracellular locations. For example, in Arabidopsis, a collection of *cameleon* constructs adapted for plants and specifically targeted to the cytoplasm, nucleus, or plasma membrane was developed (Krebs et al., 2012). Therefore, plant *cameleons* are nowadays powerful tools to study Ca^2+^ oscillations in plant cells.

In this work, we assayed two different protocols in order to optimize carrot transformation and develop for the first time transgenic lines expressing *cameleon* probes. We produced three carrot lines expressing plasma membrane-targeted *cameleons* and used them, together with their wild type counterparts, to induce somatic embryogenesis, characterize the different stages of the process, study the dynamics of callose deposition, image Ca^2+^ distribution in living cells during embryo induction and development and test the effect of applying different Ca^2+^ chemical modulators at different times, principally during the first stages of embryo induction. Our results show that Ca^2+^ is tightly associated to the embryogenic fate of specific cells. Besides, Ca^2+^ homeostasis can be altered to modulate embryo production. Together, these results help to improve and understand this fascinating *in vitro* process.

## Materials and methods

### Plant material

Commercial seeds of carrot (*Daucus carota)* cv. Nantese 5 from Semillas Batlle S.A. were used for transformations, induction of somatic embryogenesis and modulation of Ca^2+^ homeostasis. Transgenic seedlings developed from carrot transformation expressing the PM-YC3.6-LTI6b *cameleon* construct were used for Ca^2+^ visualization by confocal microscopy. Carrot seeds were surface-sterilized in two consecutive cycles of 15 minutes in 70% ethanol + 0.1% triton solution, 15 minutes in 50% bleach solution and three rinses in distilled sterile water. Seeds were kept in solid MS2% medium (**Table 1**) for 7-10 days at 25°C in darkness.

**Table 1:** Composition of the seed germination and plant *in vitro* culture media used for the carrot transformation protocol D.

	Solid MS2%	Liquid MS2%	Co-culture D	MI	MII	MIII	Immobilization
MS (g/L)	4.6	4.6	4.6	4.6	4.6	4.6	9.2
Sucrose (%)	2	2	2				4
Myo-inositol (%)			0.05				
2,4-D (mg/L)			1	0.5	0.25		
Carbenicilin (mg/L)				500	500	500	
Plant agar (%)	0.8		0.8	0.8	0.8	0.8	
pH	5.8	5.8	5.8	5.8	5.8	5.8	5.8

MS, Murashige and Skoog basal medium + vitamins (Murashige and Skoog, 1962). All basal media and other medium components were purchased from Duchefa (Netherlands).

### Plant transformation and regeneration

Two *cameleon* constructs were used for carrot transformation: YC3.6-Bar and PM-YC3.6-LTI6b, coding for *cameleon* fusion proteins targeted to the cytoplasm and the cytosolic side of the plasma membrane (Krebs et al., 2012). Both *cameleon* plasmids were kindly provided by Dr. Jörg Kudla and described in detail in (Krebs et al., 2012). *Escherichia coli* One Shot™ ccdB Survival™ 2 T1R Competent Cells were transformed with YC3.6-Bar and PM-YC3.6-LTI6b plasmids respectively. The *Agrobacterium tumefaciens* strain LBA4404 carrying the helper *vir* plasmid pAL4404 (ElectroMAX™ *A. tumefaciens* LBA4404 Cells, Invitrogen™) was transformed with the YC3.6-Bar and PM-YC3.6-LTI6b plasmids respectively following provider instructions. Transformed cells were plated in YM solid medium supplemented with 50 mg/L rifampicin and appropriate bacterial selective agents: 50 mg/L kanamycin for YC3.6-Bar and 100 mg/L spectinomycin and 100 mg/L streptomycin for PM-YC3.6-LTI6b.

Two transformation protocols were assayed, namely protocol D (Gonzalez-Calquin and Stange, 2020) and protocol E (Hardegger and Sturm, 1998). Protocol D started pre-culturing 2 cm-long hypocotyl explants for 3 days at 25°C in darkness in Co-culture D medium (**Table 1**). Explants were incubated for 15 min in *Agrobacterium tumefaciens* suspensions prepared in 4.6 g/L MS medium at a final OD_600_ of 0.2. Incubation was performed in petri dishes. During the process, small wounds were performed in the explants with a scalpel to facilitate the penetration of bacteria in the explants. Then, explants were deposited in sterile paper to remove the excess of bacterial inoculum. Explants were placed in Co-culture D medium and incubated for 3 days at 25°C in darkness. After that, explants were transferred to MI medium (**Table 1**) and plates were kept at 25°C in darkness. Two weeks later, explants were transferred to MII medium (**Table 1**) and incubated for at least one month, refreshing the medium every two weeks. MII plates were kept at 25°C in a 16/8 h photoperiod. When developing embryos were visible and detached from the explant, explants were transferred to plates with MIII medium (**Table 1**) supplemented with 5 mg/L BASTA for explants transformed with YC3.6-Bar and with 50 mg/L kanamycin for explants transformed with PM-YC3.6-LTI6b, and kept at 25°C and 16/8 h photoperiod. Medium was refreshed monthly.

For protocol E, 2 cm hypocotyl explants were obtained from 7-10 day-old seedlings. Inoculation was assayed under two different conditions: dipping the explants for few minutes in the suspension preparation or applying three cycles of vacuum (5 minutes each) to the explants. In both cases, after inoculation, explants were incubated in Co-culture E medium (**Table 2**) for 3 days in darkness at 25°C. Excess of bacteria was washed with liquid basic GB5 medium and explants were incubated in Selection 1 medium (**Table 2**) for two weeks at 25°C and 16/8 h photoperiod. Then, explants were transferred to Selection 2 medium (**Table 2**) and incubated for six weeks at 25°C and 16/8 h photoperiod. Medium was refreshed every two weeks. For plant regeneration, an adaptation of the (Overvoorde and Grimes, 1994) protocol was followed. Greenish calli were transferred to Embryogenesis Induction Selective Medium (EISM; **Table 2**) and kept for at least eight weeks at 25°C and 16/8 h photoperiod, refreshing plates every two weeks. Portions of approximately 0.2 g of resistant calli were transferred to 100 mL flasks containing 50 mL of liquid EISM (excluding plant agar), and kept for one week in a rotary shaker (140 rpm) at 25°C in darkness. Then, liquid cultures were filtered through a 41 µm nylon filter. Cells were recovered with 50 mL of liquid MS2% medium (**Table 1**) supplemented with 500 mg/L carbenicilin and transferred to 100 mL flasks. Flasks were kept in a rotary shaker at 25°C in darkness for at least one week. 1 mL aliquots of cultures were transferred to plates with solid MS2% medium (**Table 1**) and incubated at 25°C and 16/8 h photoperiod. When embryos germinated into seedlings, a portion of cotyledon was used for genotyping with the primers pUBQ 5’TTCACCGCCTTAGCTTTCTCG (forward) and CFP5’ 5’GCACGACTTCTTCAAGTCCGC (reverse).

**Table 2:** Composition of the plant *in vitro* culture media used for transformation protocol E and for induction of somatic embryogenesis.

	Co-culture E	Selection 1	Selection 2	EIM	EISM
GB5 (g/L)	3.2	3.2	3.2	3.2	3.2
Sucrose (%)	3			3	
2,4-D (mg/L)				0.2	0.1
Carbenicilin (mg/L)		500	500		500
Geneticin (mg/L)			10		10
NAA (mg/L)	1	1	1		
BAP.(mg/L)	0.5	0.5	0.5		
Plant agar (%)	0.8	0.8	0.8	0.8	0.8
pH	5.8	5.8	5.8	5.8	5.8

GB5, Gamborg B5 basal medium + vitamins (Gamborg et al., 1968); EIM, Embryogenesis Induction Medium; EISM, Embryogenesis Induction Selective Medium; BAP, 6-benzylaminopurine; NAA, naphthaleneacetic acid. Geneticin is a kanamycin derivative. All basal media and other medium components were purchased from Duchefa (Netherlands).

### Somatic embryogenesis

Induction of carrot somatic embryogenesis was performed as previously described (Overvoorde and Grimes, 1994), with some modifications. Two-cm hypocotyl explants, directly taken from *in vitro*-growing transgenic seedlings, were cultured in solid Embryogenesis Induction Medium (EIM; **Table 2**), for at least 40 days at 25°C in darkness, until abundant calli were clearly developed from the explants. Then, 0.2 g of the developed calli were disaggregated in 100 mL flasks containing 50 mL of liquid EIM (excluding plant agar) and kept in a rotary shaker for 7 days at 140 rpm, 25°C in darkness. Then, cell cultures were filtered through 41 µm nylon filters and the filters were transferred to sterile glass flasks. Retained cells were washed with 50 mL liquid MS2% medium (**Table 1**), transferred to a new flask and incubated for 7 days in agitation in a rotary shaker at 140 rpm, 25°C in darkness. Aliquots of 1 mL of embryogenic cell cultures were transferred to 3-cm sterile culture dishes and kept at 25°C in darkness and no agitation for 30 days. In immobilized carrot cultures, aliquots of 200 µL of immobilization medium (**Table 1**) were mixed with 200 µL of embryogenic cell cultures in 3 cm culture dishes. Then, 200 µL of autoclaved 1.5% liquid low melting point agarose (SeaPlaque, Duchefa), pre-warmed at 60°C, were gently pipetted into the dishes. Open dishes were incubated on ice for 5 min until the culture medium gelified, covered with the lid, sealed and kept upside down (immobilized cells up) to avoid condensation on immobilized cells. Dishes were kept at 25°C either in darkness or in a 16/8 h photoperiod. For all carrot cultures, the proportion of embryos was calculated 30 days after culture initiation by counting the number of embryos (considered as bipolar structures with a clear heart-shaped, torpedo or cotyledonary morphology) and dividing it by the total number of structures (non-growing clumps, calli and embryos) counted. For both embryo and total counts, the field counting method was used (Camacho-Fernández et al., 2018). At least 200 structures per dish were counted.

### Chemical treatments

For the chemical treatments, stocks of CaCl_2_, ionophore A23187, ethylene glycol-bis(β-aminoethyl ether)-N,N,N′,N′-tetraacetic acid (EGTA) and N-(6-Aminohexyl)-5-chloro-1-naphthalenesulfonamide hydrochloride (W-7) were prepared following the guidelines provided by the manufacturer. Embryogenic cell cultures from liquid MS2% flasks, containing mostly proembryogenic masses and cell clumps, were inoculated into 3-cm culture dishes. To allow for comparisons among stages and concentrations for each chemical, equal aliquots (1 mL) of the same embryogenic cell culture were inoculated, so the initial amounts of proembryogenic masses/cell clumps were the same in all cases. Then the different chemicals, at different concentration (as described in Results), were applied during the first three days, during the first seven days, or continuously (for 30 days). Control plates were prepared with the proper concentrations of the solvent used to dilute the corresponding compound. At least three different repeats were performed for each chemical, concentration and exposure time applied. In all cases, plates were kept at 25°C in darkness and no agitation. For the cultures with three and seven days of exposure, experimental plates with the chemical (and their corresponding control) were centrifuged (800 rpm, 4 min, 25°C in a refrigerated Eppendorf Centrifuge 5804R with A-4-44 rotor) to wash the compound. The proportion of embryos was calculated 30 days after culture initiation as described for somatic embryogenesis cultures.

### 2-deoxy-D-glucose and callose staining

To inhibit callose deposition, we applied 2-deoxy-D-glucose to two different types of cultures, wild-type carrot calli disaggregated in liquid EIM, where predominantly cell clumps can be found, and one-week-old liquid EIM cultures filtered and resuspended in liquid MS2%, where proembryogenic masses can predominantly be found. One mL aliquots of each culture were poured in 3-cm sterile culture dishes and cultured for a week without (controls) and with 2-deoxy-D-glucose at 0.1, 1 and 5 mM. Incubations were performed in a rotary shaker at low speed and at room temperature. At least three different repeats were performed for each 2-deoxy-D-glucose concentration and exposure time applied. The proportion of embryos was calculated 30 days after culture initiation as described for somatic embryogenesis cultures. For visualization in the confocal microscope, control and 2-deoxy-D-glucose samples were collected in tubes, centrifuged (2 min, 8,000 rpm) and stained with 1% aniline blue for 10 min. Then, samples were washed three times with PBS 1x. Approximately 20 µL of sample were mounted in a glass slide, covered with a coverslip and observed in the confocal microscope.

### Confocal microscopy

Carrot samples stained with aniline blue were observed in a Zeiss 780 Axio Observer confocal laser scanning microscope at an excitation wavelength of 405 nm. Emission was recorded at 450 nm. Cell cultures from PCR-positive carrot plants were imaged using a Zeiss LSM880 laser scanning confocal microscope. Samples were excited at 440 nm (for CFP excitation) and 514 nm (for YFP excitation) and emission was recorded between 400 and 600 nm.

For FRET imaging of embryogenic cultures from carrot transgenic lines, embryogenic structures were mounted in a petri dish using half-strength MS medium (pH 5.7) supplemented with 0.3% agarose. Mounted samples were covered with liquid half-strength MS medium (pH 5.7) and observed in a Leica SP8-FLIMan confocal laser scanning microscope using an immersion objective. CFP was excited at 448 nm and emission was recorded between 460 and 493 nm, YFP was excited at 514 nm and recorded between 525 and 560 nm. FRET imaging was performed exciting at 448 nm and recording the emission between 525 and 560 nm. For imaging, the FRET ratio was defined as the ratio between YFP and CFP emissions (480/535 nm). Image treatment and calculation of fluorescence emission ratios were performed as previously described (Rizza et al., 2019). For all cases, Ca^2+^ levels were defined as very low, low, moderate, high or very high according to the colorimetric scale based on the FRET ratio images. Very low Ca^2+^ levels corresponded to dark blue colors, low levels to light blue, moderate to green-yellow, high to orange-red and very high levels to purple-white colors. Image analysis was performed using the FIJI software (Schindelin et al., 2012).

### Statistical analysis

Data were analyzed using the ANOVA test. Groups of significance were established according to the least significant difference (LSD) test (p ≤ 0.05). All the analyses were performed with the Statgraphics software.

## Results

### Development of *cameleon* carrot lines

**Figure 1:**
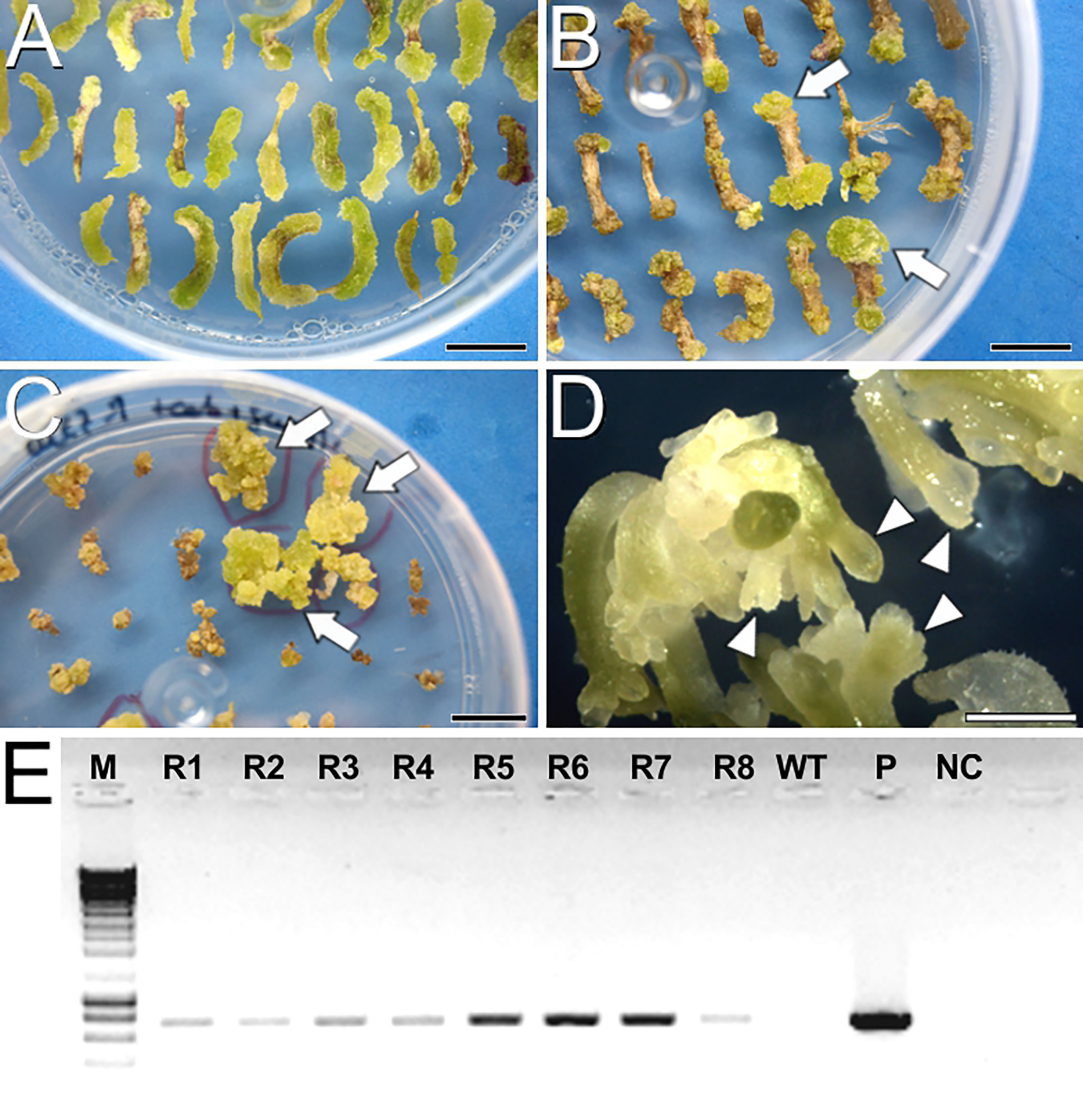
Carrot transformation and regeneration using protocol E. **(A)** Hypocotyl explants after 15 days in Selection 1 medium. **(B)** Hypocotyl explants after one month in Selection 2 medium. Arrows point to green, growing calli resistant to the selective agents. **(C)** Calli after one month in EIM with selective agents. **(D)** Full embryos developed in solid MS2% medium. Arrowheads point to developing somatic embryos. **(E)** Agarose electrophoresis gel showing PCR amplification of the transgene. From left to right, M, DNA molecular weight marker; R1-R8, Eight plantlets regenerated from the four resistant calli; WT, Wild type plant; P, PM-YC3.6-LTI6b plasmid; NC, PCR negative control. Bars: **(A-C)**: 1 cm; D: 1 mm.

To produce *cameleon* carrot lines, we first tested two carrot transformation protocols, namely D (Gonzalez-Calquin and Stange, 2020) and E, which is a combination of previously reported transformation (Hardegger and Sturm, 1998) and regeneration protocols (Overvoorde and Grimes, 1994). For protocol D (**Supplementary Figure S1**), we inoculated an average of 184 ± 69 hypocotyl explants per assay transformed with the YC3.6-Bar construct, and 163.5 ± 28 explants per assay transformed with the PM-YC3.6-LTI6b construct. YC3.6-Bar explants did not produce any embryo, and PM-YC3.6-LTI6b produced embryos that eventually died upon transference to media with kanamycin as selective agent. Kanamycin has been reported to inhibit cell growth and prevent embryo formation in some carrot cultivars (Hardegger and Sturm, 1998). Since this seemed to be our case, we focused on protocol E for transformation of explants with the PM-YC3.6-LTI6b construct using geneticin, a kanamycin derivative, as selective agent. An average of 196.67 ± 33.3 explants per assay were inoculated. After two weeks in Selection 1 medium, most of the explants developed greenish calli on their surface and/or at their ends (**Figure 1A**). After one month in Selection 2 medium (**Figure 1B**), clear differences were observed between calli resistant (green) and sensitive to the selective agents (whitish or brown). Green calli were transferred to EIM supplemented with selective agents and along four rounds of medium refreshment during two months, some turned brown and arrested growth while others kept green and growing (**Figure 1C**). Green and growing, potentially transformed calli were cultured in liquid EIM and, upon transference to MS medium, they eventually produced fully developed embryos (**Figure 1D**). Finally, eight full seedlings from four resistant embryo-producing calli were regenerated and all of them tested positive for the construct by PCR analysis (**Figure 1E**). Cell cultures from one PCR-positive plant per line (four plants in total) were imaged at the confocal microscope to check for FRET performance (**Supplementary Figure S2**), confirming the successful production of three different PM-YC3.6-LTI6b *cameleon* lines using protocol E. Two of them showed an equivalent and stronger signal for both CFP and YFP. We focused on the use of these two lines for all FRET experiments.

### Somatic embryogenesis

**Figure 2:**
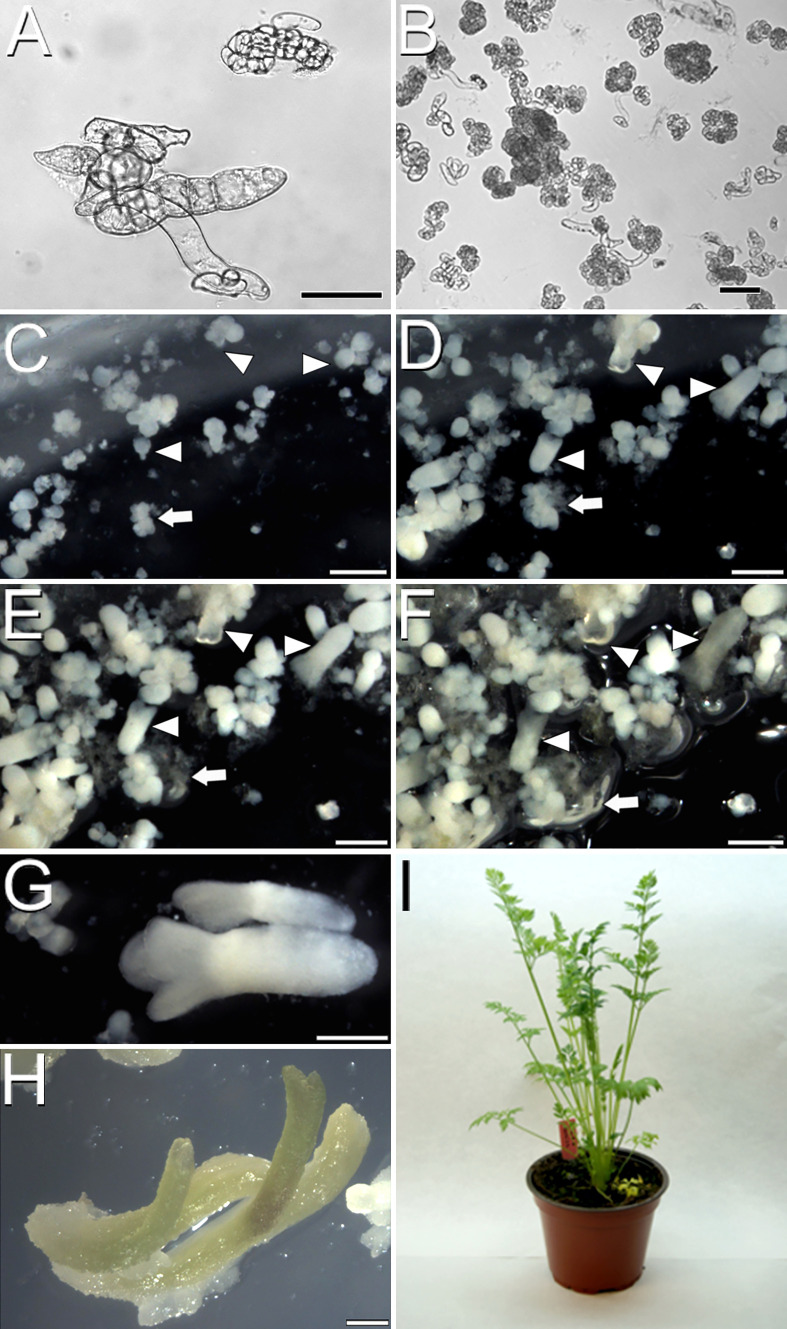
Carrot somatic embryogenesis. **(A)** Cell clumps after one week in liquid EIM. **(B)** Compact embryogenic masses after one week in liquid MS2%. **(C-F)** Time-lapse images of an immobilized culture one week **(C)**, two weeks **(D)**, three weeks **(E)** and one month after immobilization **(F)**. Arrowheads point to individual developing embryos progressing through different developmental stages. Arrows point to an individual proliferating callus mass. **(G)** Fully developed embryo after three weeks in liquid MS2%. **(H)** Embryos germinated in solid MS2%. **(I)** Acclimatized adult carrot plant regenerated from somatic embryos. Bars: **(A, B)**: 50 µm; C-H: 500 µm.

Once developed the *cameleon* carrot lines, we induced somatic embryogenesis using both liquid and immobilized cultures to track changes during the process. After 40 days of culture in solid EIM, carrot hypocotyls developed calli that were disaggregated in liquid EIM under agitation, producing isolated cell clumps, defined as irregularly shaped cell clusters with weak cell adhesion (**Figure 2A**). Upon culture in liquid MS2% medium with agitation, some cell clumps proliferated as embryogenic masses, defined as compact cell aggregates with higher structural order, showing the first signs of differentiation into early (globular) embryo-like structures (**Figure 2B**). In immobilized cultures, we tracked the progression from globular-like to torpedo embryos in one month (**Figures 2C-F**). Time-lapse imaging also revealed the developmental asynchrony of the different structures, since from an initial population enriched in embryogenic masses (**Figure 2C**), embryos at all possible developmental stages were observed after one month (**Figure 2F**, arrowheads), together with irregularly shaped, disorganized callus-like masses (**Figure 2F**, arrow) coming from some of the initial masses.

In order to optimize the process, we evaluated the possibility of reducing the stage of culture with agitation from two to just one week and directly moving the cell clumps to liquid MS2% medium without agitation or to solid, immobilization medium. In all cases, the second week of agitation was found essential to increase and accelerate the production of embryogenic masses and to reduce the formation of disorganized callus masses (**Supplementary Figure S3**). Embryo progression was higher and faster in liquid cultures, where mature, cotyledonary embryos ready to germinate (**Figure 2G**) were produced in just three weeks of culture. Irrespective of the culture method used, embryos germinated when transferred to solid MS2% (**Figure 2H**) and became entire, adult plants upon acclimatization and transference to soil (**Figure 2I**). In conclusion, both liquid and immobilized cultures are useful to produce carrot somatic embryos. Immobilized cultures are convenient for experiments where embryos must develop in fixed positions, but liquid culture is faster and more efficient. In both cases, somatic embryos and callus masses are formed, and the second week in agitation is essential for a proper cell clump-to-embryo conversion.

### Distribution of Ca^2+^ during carrot somatic embryogenesis

**Figure 3:**
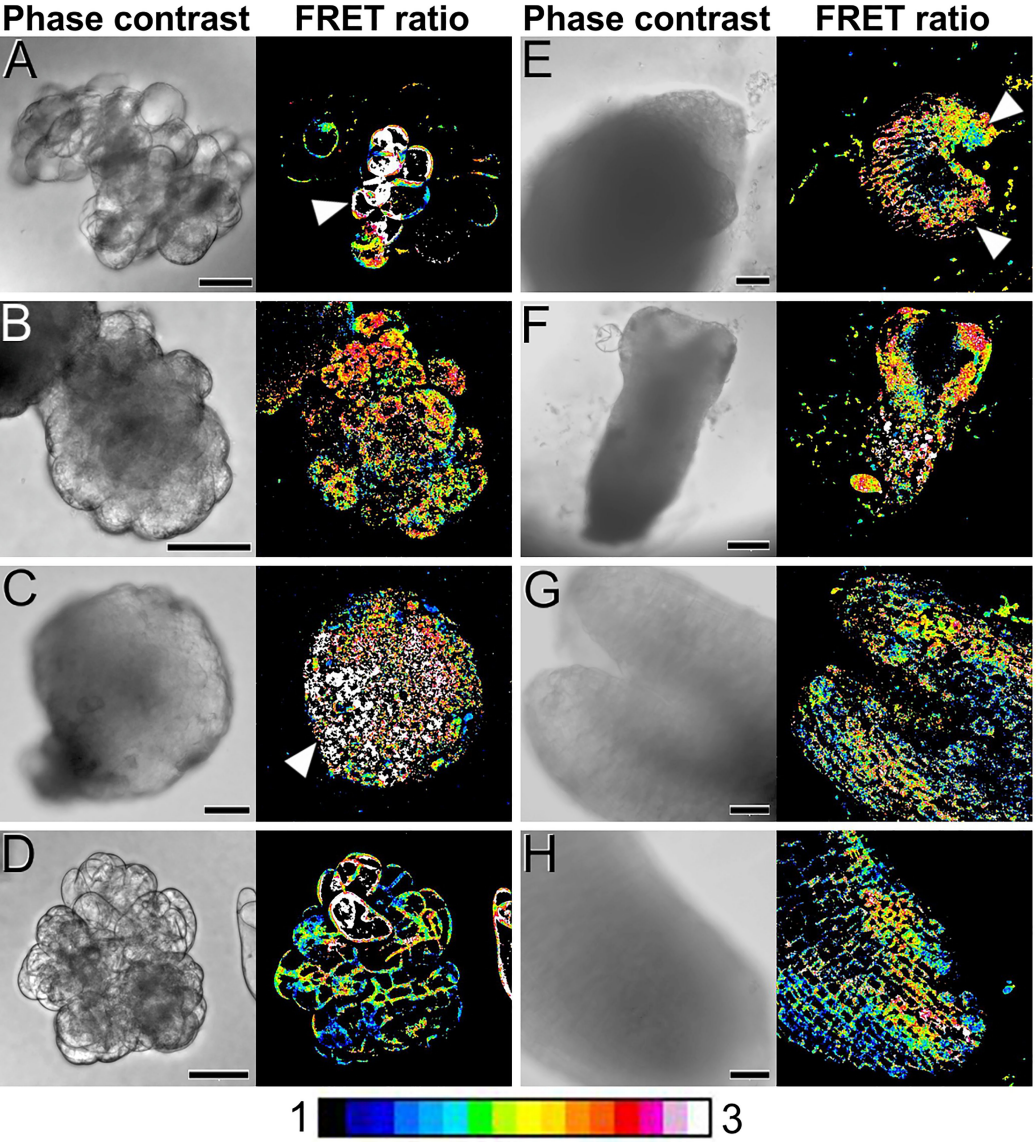
FRET imaging of Ca^2+^ levels during carrot somatic embryogenesis in PM-YC3.6-LTI6b *cameleon* lines. Each pair of images shows the same field imaged by phase contrast (left) and FRET ratio (YFP/CFP emissions; right). The LUT bar displays the false coloration of FRET ratios. **(A)** One-week-old cell clumps. Note the higher Ca^2+^ levels of the cells at the left side (arrowheads). **(B)** Cell clump transforming into an embryogenic mass. Note the higher Ca^2+^ levels of the cell mass at the left side (arrowhead). **(C, D)** Two-weeks-old compact, globular embryo **(C)** and callus mass **(D)**. Arrowhead in C points to a pole with higher Ca^2+^ levels. **(E)** Three-weeks-old heart-shaped embryo. Arrowheads point to the cotyledon primordia. **(F)** Torpedo embryo. **(G, H)** Shoot **(G)** and root **(H)** apical regions of a cotyledonary embryo. Bars: **(A-E, G, H)**: 40 µm; F: 100 µm.

Next, we used our *cameleon* carrot lines to study, using FRET imaging, how Ca^2+^ distributes in the different structures formed during the process of somatic embryo induction and development (**Figure 3**; **Supplementary Figure S4**). During the first week of culture, just induced cell clumps (**Figure 3A**) presented irregular morphologies, with loosely attached cells and in general low-moderate Ca^2+^ levels in the plasma membranes of all their cells. However, there were defined regions, generally at one end of the structure, that presented higher Ca^2+^ levels (**Figure 3A**, arrowhead). These regions developed into compact, globular embryogenic cell masses that maintained high Ca^2+^ levels (**Figure 3B**). After two weeks of culture, compact, globular embryos increased their size and their Ca^2+^ levels increased to high or very high. The distribution, however, was not uniform, concentrating towards one pole of the embryo (**Figure 3C**). At this stage, non-embryogenic callus masses clearly differed from globular embryos in their Ca^2+^ levels, which were typically low-moderate for most of their cells, with few, exceptional cells with higher levels (**Figure 3D**), in a pattern similar to that of cell clumps. In three-week-old cultures, differentiation of globular embryos into heart-shaped somatic embryos coincided with a non-uniform distribution of Ca^2+^, which concentrated at the periphery of the embryo, coinciding with the protodermal layer, and especially in the apical regions (shoot apical meristem and cotyledons, **Figure 3E**). In elongating torpedo embryos, Ca^2+^ clearly concentrated in the actively growing cotyledons and the shoot apical meristem, which presented high Ca^2+^ levels all along their inner regions (**Figure 3F**). The root meristem region, however, presented low Ca^2+^ levels. In developed cotyledonary embryos, moderate-high Ca^2+^ levels principally concentrated in the inner mesophyll region of the cotyledons (**Figure 3G**) and the root meristem (**Figure 3H**), being low or very low in all other regions of the embryo. Together, these results show that Ca^2+^ levels are highly dynamic during the different stages of somatic embryogenesis, concentrating in the proliferating cells and embryogenic structures, and in the actively growing regions of the developing somatic embryo. These results point to a role of Ca^2+^ in the induction of somatic embryogenesis and the development of somatic embryos.

### Distribution of callose during carrot somatic embryogenesis

**Figure 4:**
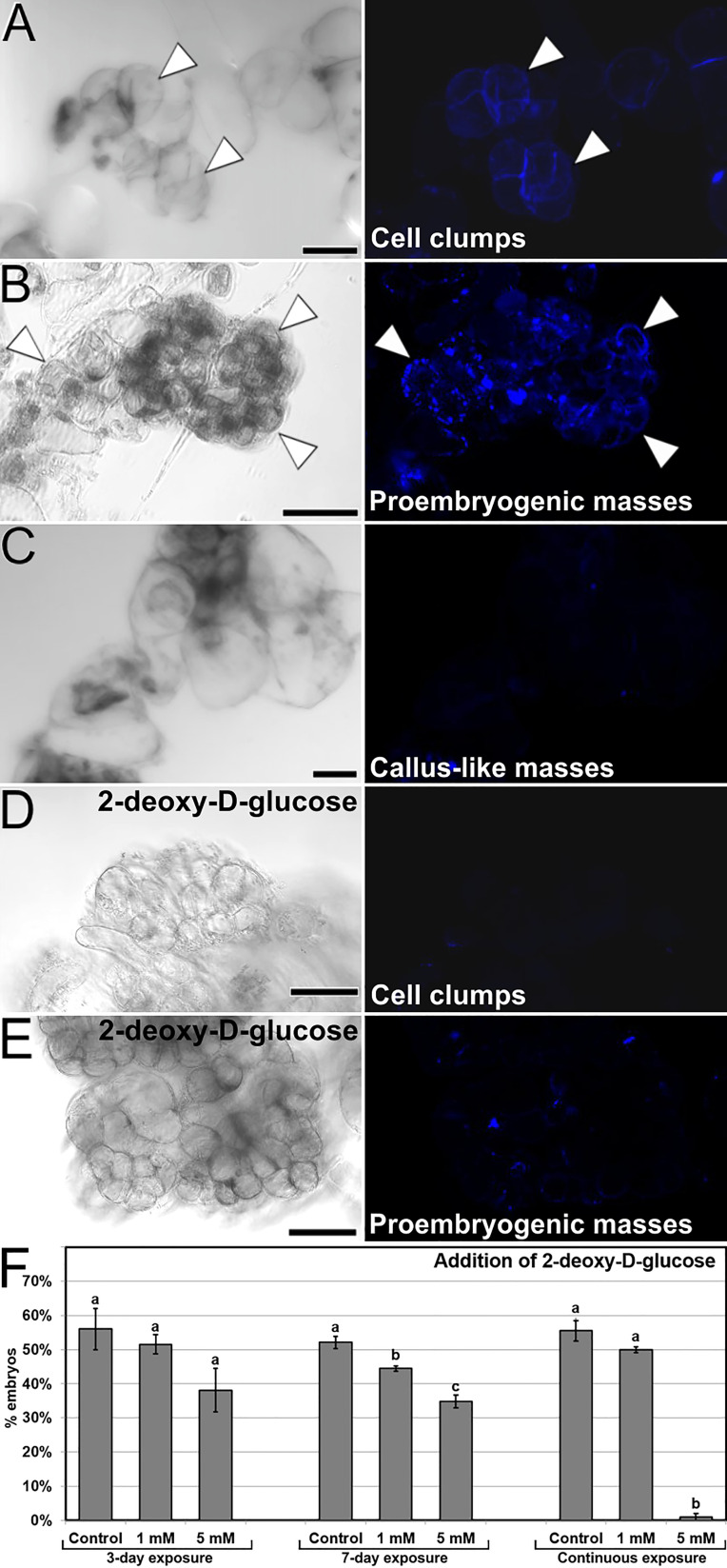
Callose deposition in cultured embryogenic structures. Each pair of **(A-E)** images shows the same field of aniline blue-stained samples imaged by phase contrast (left) and fluorescence (right). **(A, B)** Small clusters of callose-positive cells in cell clumps **(A)** and proembryogenic masses **(B)**. Note the intense aniline blue staining in the cell wall region of specific cells (arrowheads). **(C)** Irregular callus mass with loosely connected cells. **(D, E)** Small cell clump **(D)** and proembryogenic mass **(E)** from cultures with 5 mM 2-deoxy-D-glucose. **(F)** Modulation of callose deposition by the addition of 2-deoxy-D-glucose at different concentrations and 3-day, 7-day and continuous exposures. The effects of the treatments are expressed as percentages of embryos produced (% embryos). Letters represent significant differences with respect to their respective controls according to the LSD test. Bars: **(A, B, D, E)**: 40 µm; C: 80 µm.

We stained embryogenic cultures with aniline blue to study the pattern of callose deposition. Abundant callose accumulation at the cell wall region was found surrounding particular cells in the form of a continuous layer, generally in cells at one end of small clumps of loosely connected cells (**Figure 4A**). This pattern of permanent callose deposition in a layer surrounding the cell does not correspond with its conventional, transient role during plant cytokinesis (Thiele et al., 2009). Thus, callose deposition must be related to embryogenesis induction. The shape, position and size of the aniline blue-positive cells and the cells with high Ca^2+^ levels (**Figure 3A**) was similar, indicating a relationship between increased Ca^2+^ levels and callose deposition. Similar callose-positive cells were found at specific regions of small proembryogenic masses, before embryo differentiation (**Figure 4B**). In callus-like masses, where Ca^2+^ levels were mostly low or moderate (**Figure 3D**), the levels of callose staining were much lower, almost negligible (**Figure 4C**). These results showed that during the very first stages of induction of somatic embryogenesis, cultured embryogenic carrot cells develop a callose layer around them, while non-embryogenic callus-like masses do not develop such layer.

In order to elucidate the role of callose in the initial stages of the process, we added 2-deoxy-D-glucose, an inhibitor of callose biosynthesis, at different concentrations in the culture medium during the first 3 and 7 days of culture, as well as during all the culture time. With 1 mM and principally with 5 mM 2-deoxy-D-glucose, we observed that cells of both cell clumps (**Figure 4D**) and small proembryogenic masses (**Figure 4E**) showed almost no aniline blue staining, indicating a lack of callose deposition in their cell walls. In order to study the relevance of the absence of callose in cell walls of these structures, we quantified the somatic embryogenesis response of explants treated with different concentrations and application times of 2-deoxy-D-glucose. The observed lack of callose correlated with the quantification of the embryos produced (**Figure 4F**), which had a significant negative effect when applied at 5 mM during both the first 7 days and continuously, where embryo production was almost null. In conclusion, in addition to calcium, callose deposition in the cell walls during the stages of embryo induction and differentiation has a critical role in carrot somatic embryogenesis.

### Modulation of Ca^2+^ homeostasis

**Figure 5:**
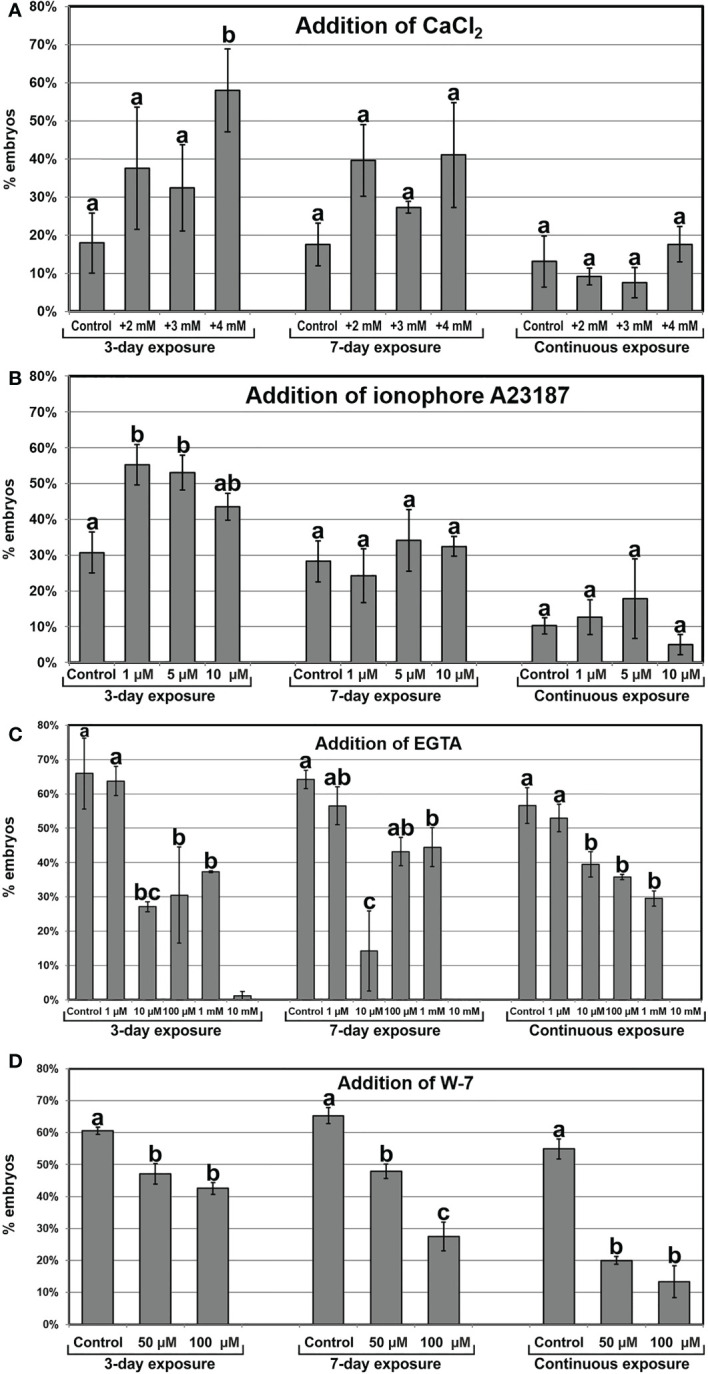
Modulation of intracellular Ca^2+^ homeostasis by the addition of CaCl_2_ **(A)**, the Ca^2+^ channel ionophore A23187 **(B)**, EGTA, a Ca^2+^ chelator **(C)**, and W-7, a CaM antagonist **(D)** at different concentrations and 3-day, 7-day and continuous exposures. The effects of the treatments are expressed as percentages of embryos produced (% embryos) ± standard error. At least three different repeats were performed for each chemical, concentration and exposure time applied. Data were analyzed using the ANOVA test and groups of significance were established according to the least significant difference (LSD) test (p ≤ 0.05). Different letters represent significant differences with respect to their respective controls according to the LSD test.

**Figure 6:**
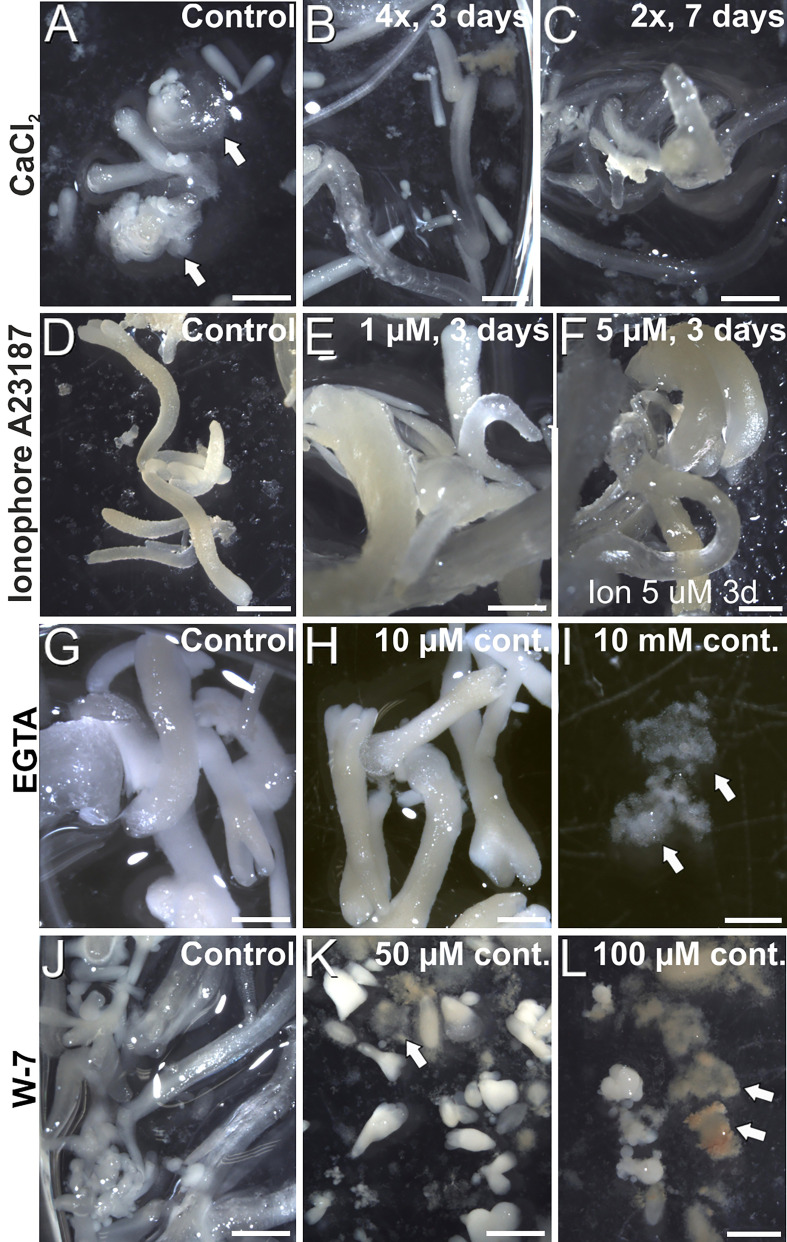
Carrot somatic embryos produced in cultures with added CaCl_2_ **(A-C)**, ionophore A23187 **(D-F)**, EGTA **(G-I)** and **(J-L)** at different concentrations and durations, as described in the images. Arrows point to disorganized callus masses. Bars: 1 mm.

Once established the relationship between increased Ca^2+^ levels and induction of somatic embryogenesis, we performed a pharmacological study to modulate the intracellular Ca^2+^ levels. We treated embryogenic liquid carrot cultures with different chemicals known to interfere with intracellular Ca^2+^ levels and calculated the percentage of embryos produced by each treatment after 30 days of culture. First, we increased the levels of available Ca^2+^ by adding increased CaCl_2_ concentrations (2, 3 and 4 mM) to the culture medium (**Figure 5A**), and compared embryo production with that of control cultures. When applied during the first 3 days of culture, +4 mM CaCl_2_ produced significantly more embryos (3-fold) than the control. In these conditions, addition of increased CaCl_2_ levels also reduced the occurrence of disorganized calli and accelerated embryo growth, producing larger and elongated embryos (**Figures 6A-C**). A 7-day and a continuous exposure during all culture stages to increased CaCl_2_ levels did not produce any significant change in embryo production (**Figure 5A**). Together, these observations indicate that Ca^2+^ modulation is effective to increase embryo induction and growth during the first stages of the process. Next, we applied ionophore A23187, a plasma membrane-intercalating channel that allows Ca^2+^ to freely cross it, increasing Ca^2+^ influx and thereby altering intracellular gradients (Ge et al., 2007). The application of 1 and 5 µM ionophore A23187 during the first three days of culture significantly increased the production of somatic embryos (**Figure 5B**). As for the addition of CaCl_2_, the conditions that produced more embryos also promoted an accelerated embryo growth, producing larger and more mature, sometimes germinating embryos (**Figures 6D-F**). Applications during the first 7 days or continuously did not show significant differences with respect to controls without the chemical. Together, these results confirmed the role of Ca^2+^ during the first stages of somatic embryo induction and, most importantly, showed that an increase of Ca^2+^ levels can be beneficial for embryo induction and development.

Next, we studied the effect of reducing intracellular Ca^2+^ levels with EGTA, a highly specific Ca^2+^ chelator capable of freely diffusing through the plasma membrane. With the range of EGTA concentrations used (**Figure 5C**), we observed a significant reduction of embryo production, which was completely inhibited at the highest concentration (10 mM). These effects were similar for the 3-day, 7-day and continuous EGTA application. The reduction was accompanied by a dose-dependent delay in embryo growth and the increased occurrence of disorganized callus masses, which were the only morphogenic structures observed at the highest EGTA concentration (**Figures 6G-I**). Thus, Ca^2+^ chelation dramatically affects somatic embryogenesis, reducing the proportion of somatic embryos induced and their further development. Finally, we applied W-7, a CaM antagonist (**Figure 5D**). For all the concentrations tested, inhibition of CaM by W-7 significantly reduced the production of somatic embryos. The extent of the reduction was dependent on the dose used and the duration of the treatment. In addition, embryos exposed to W-7 were less developed than control embryos of the same age, suggesting that W-7 delays embryo development (**Figures 6J-L**). W-7 also promoted the conversion of embryos into large, disorganized calli. Thus, interference of Ca^2+^-CaM binding by W-7 negatively affects embryo production and development, producing an effect very similar to that observed with the Ca^2+^ chelator. In summary, altering Ca^2+^ signaling by reducing their levels or by interfering with CaM has a significantly negative impact on embryogenesis induction.

## Discussion

Artificial, *in vitro* embryogenesis systems and, in particular, somatic embryogenesis systems such as the one we present here are very useful tools first in plant biotechnology, and second in basic plant biology to study different aspects of embryo development. The *in vitro* development of “naked” embryos facilitates their visualization and study, which is very difficult during zygotic embryogenesis due to the different tissue layers that surround the minute embryo during the first stages of its development and preclude its correct visualization and/or isolation. We report on the generation of three functional *cameleon* carrot lines. This is, to the best of our knowledge, the first work to develop *cameleon*-expressing carrot lines. These lines open the way to study the role of Ca^2+^ in living carrot cells in many different *in vivo* and *in vitro* processes. We used them here to study the role of Ca^2+^ during this process, as described next.

### Mechanical stress is beneficial for carrot somatic embryogenesis

Different protocols for carrot somatic embryogenesis implement a step of culture in liquid medium under an agitation regime (Timmers et al., 1989; Overvoorde and Grimes, 1994). In an attempt to optimize the process, we reduced such step to one week. As a result, the occurrence of embryos was significantly reduced and callus formation was increased. We hypothesize that, in addition to a better access to medium components, culture agitation provides cells with a mild and prolonged mechanical stress that would be beneficial for induction of *in vitro* embryogenesis. In another morphogenic process of *in vitro*-induced embryogenesis such as microspore embryogenesis, high-speed centrifugation for 30 min was able to induce tobacco microspores towards embryogenesis (Tanaka, 1973). Even shorter and milder centrifugations were effective for chickpea microspores to increase embryo yield and reduce callus occurrence (Grewal et al., 2009). We observed a similar effect in carrot somatic cell cultures with agitation, which can be considered a milder and prolonged form of centrifugal stress. Additional evidence for this comes from the fact that, for all the chemicals used to modulate Ca^2+^ levels (**Figure 5**), the controls of the three and seven-day exposure to the chemical, which include a centrifugation step to wash the medium, always showed higher percentages of embryo production than the control of continuous exposure to the compound, without centrifugation. It was postulated that centrifugation would alter the auxin-to-cytokinin ratio, thereby facilitating embryo induction (Tanaka, 1973). Instead, we believe that, for both microspore and somatic embryogenesis, mechanical stress (centrifugal in this case) would be a source of abiotic stress that, alone or combined with others, may trigger the induction of *in vitro* embryogenesis. This is a well known effect in microspore embryogenesis systems (Seguí-Simarro et al., 2021), where the combination of different stresses has increased beneficial effects in embryo production and callus avoidance (Grewal et al., 2009).

### High Ca^2+^ levels are markers of embryogenic induction and embryo patterning

Carrot embryogenic cultures typically include cell clumps that become either disorganized callus masses or embryo-producing, proembryogenic masses. FRET imaging in living cultures revealed that cell clumps show in general low or moderate Ca^2+^ levels, except for some discrete cells where Ca^2+^ levels are higher. Similar results were previously observed using fluorescent probes (Timmers et al., 1989). These regions are then transformed into proembryogenic masses that seem not to be just disorganized structures but organized embryo precursors, since high Ca^2+^ levels are typically found in cells of discrete regions at fixed positions. This is also supported by the polarized localization of CaM in particular regions of these masses (Timmers et al., 1989) and by the similarities between the protein patterns and gene expression programs of carrot somatic embryos and their precursors, the proembryogenic masses (Sung and Okimoto, 1981; Wilde et al., 1988). In turn, cells of undifferentiated callus masses keep in general low or moderate Ca^2+^ levels and never produce embryos. Thus, each structure has different Ca^2+^ patterns that seem associated to their embryogenic competence from their very beginning, with high levels in the cells of embryogenic nodes that will become embryos. This is in agreement with Ca^2+^ dynamics in other *in vitro* embryogenesis systems. In *Brassica napus*, microspores ready for embryogenesis induction and immediately after induction show high levels of intracellular Ca^2+^ (Rivas-Sendra et al., 2017; Rivas-Sendra et al., 2019). Indeed, Ca^2+^ levels were related to the embryogenic competence of microspores of different species (Rivas-Sendra et al., 2017; Rivas-Sendra et al., 2019). Therefore, Ca^2+^ would be an early marker of embryogenic fate not only for carrot somatic embryogenesis, but in general for *in vitro* embryogenesis processes.

In our living carrot cultures, Ca^2+^ gradients were observed during the development of carrot somatic embryos from the globular stage (**Figure 3C**). Upon transitioning from radial to bilateral symmetry in heart-shaped and torpedo embryos, high Ca^2+^ levels delineated the differentiating protoderm first (**Figure 3E**) and then the shoot apical meristem and cotyledons (**Figure 3F**). In mature, cotyledonary embryos, high Ca^2+^ levels marked the differentiating root meristem and the inner, mesophyll region of the cotyledons of newly formed somatic embryos (**Figures 3G, H**). An RNA-seq comparative study between the transcriptomes of somatic embryo and callus cells in Arabidopsis revealed that embryogenic cells are transcriptionally rather than metabolically active (Magnani et al., 2017). These changes involved rearrangements at the subcellular level and chromatin remodeling, repression of root meristem genes, and activation of pathways involved in shoot patterning and polarized cell growth (Magnani et al., 2017). It is interesting to note that the spatial pattern of gene expression depicted by this transcriptomic study matches with the spatial distribution of Ca^2+^ we observed during the development of carrot somatic embryos. This pattern is also similar to the reported distribution of CaM, polarized in the early somatic embryo and highly increased at the cotyledons of maturing embryos (Timmers et al., 1989). We also showed the direct relationship between somatic embryogenesis and CaM with the use of W-7, a CaM antagonist that consistently produced a dose and time-dependent decrease of embryo percentages (**Figure 5D**), and a delay and disorganization of embryo development, which in some cases reverted to calli (**Figures 6J-L**). Together, these results make reasonable to propose a direct relationship between Ca^2+^-CaM signaling and embryogenesis induction and activation of the large transcriptional remodeling undergone by somatic cells to become embryos.

### Embryogenic cells are defined by the development of a callose layer that is essential for embryo induction

We showed that a callose layer is created around specific cells of cell clumps and small proembryogenic masses which share a number of features with the embryo-precursor cells that present high Ca^2+^ levels. Although we were not able to co-localize callose staining and *cameleon* signal in our samples due to technical difficulties (overlapping of the aniline blue and CFP emission wavelengths), the shapes, sizes and positions of callose-positive (**Figures 4A, B**) and Ca^2+^-positive cells (**Figure 3A**) are the same, which makes us deduce a clear relationship between these two features. In carrot somatic embryogenesis it was already postulated that high Ca^2+^ levels could stimulate callose deposition to isolate embryogenic cells (Timmers et al., 1996). In line with this, we showed that callose must be deposited in the cell wall at these stages for a successful induction of embryogenesis in these cells, since callose inhibition with 2-deoxy-D-glucose inhibits the deposition of callose and reduces the percentage of embryo production. In Arabidopsis somatic embryogenesis from cotyledon nodes, it was demonstrated that callose symplasmically isolate cells of the proembryogenic domain from the surrounding tissue, in order to create a different chemical environment to establish cell totipotency and develop as somatic embryos (Godel-Jedrychowska et al., 2020). In *Brassica napus* microspore embryogenesis, the embryogenic competence of microspores was shown associated to their high Ca^2+^ levels and their parallel ability to form an osmoprotective, isolating callose layer through the activation of Ca^2+^-dependent callose synthases (Rivas-Sendra et al., 2019). Together, these findings clearly establish a link between Ca^2+^ levels and callose deposition, needed to isolate different cell types in different species as a previous requisite to reprogram them towards *in vitro* embryogenesis.

### Ca^2+^ homeostasis during the induction of somatic embryogenesis from carrot cells is plastic

The role of Ca^2+^ in both zygotic and somatic embryogenesis is widely acknowledged (Antoine et al., 2000; Ge et al., 2007; Calabuig-Serna et al., 2023), but in the case of carrot somatic embryogenesis, there have been a number of controversial reports regarding the importance of keeping Ca^2+^ homeostasis during the process. While some reports proposed that Ca^2+^ levels can be altered to increase the embryo induction rate (Jansen et al., 1990; Takeda et al., 2003), other studies suggested that such alterations would not be beneficial for embryo induction (Overvoorde and Grimes, 1994). To gain further insight into the role of Ca^2+^ in this process, we used different approaches to modulate intracellular Ca^2+^ levels. Increasing cytoplasmic Ca^2+^ availability during the first days of culture by adding extra CaCl_2_ to the culture medium or by using ionophore A23187 to open plasma membrane channels where through extracellular Ca^2+^ can enter the cytoplasm, led to higher percentages of embryo induction, accelerated embryo growth and lower callus occurrence. However, there seemed to be a limit for the direct relationship between Ca^2+^ and induction of embryogenesis, since a continuous increase of Ca^2+^ levels did not make any significant difference. This is in line with previous reports suggesting that excessive Ca^2+^ influx may have detrimental consequences on somatic embryogenesis (Takeda et al., 2003). Conversely, when the intracellular Ca^2+^ levels were reduced by chelating Ca^2+^ with EGTA, the percentage of embryos decreased in a dose-dependent manner. Thus, there is a range of Ca^2+^ concentrations where somatic embryogenesis can be modulated by altering Ca^2+^ homeostasis. A possible explanation for this could be that increased extracellular Ca^2+^ levels would create an even higher Ca^2+^ gradient (the cytoplasmic resting Ca^2+^ concentration revolves around 100 nM; (Hepler, 2005; Pirayesh et al., 2021) that would boost Ca^2+^ influx in more cells, thereby triggering the *embryogenic Ca^2+^ signature* in more cells. An alternative explanation would be that higher extracellular Ca^2+^ levels would make the plasma membrane less permeable. Although counterintuitive in principle, this effect has been documented for some cell types (Hepler, 2005). A calcium-induced impermeabilization of the plasma membrane would add to the cell isolation imparted by the callose layer, thereby increasing the probabilities for isolated carrot cells to become embryos. However, the positive effect observed with the addition of CaCl_2_ was similar to that of ionophore A23187, a Ca^2+^ channel that increases Ca^2+^ influx (Ge et al., 2007). Thus, it seems that, at least in carrot, adding increased Ca^2+^ concentrations to the culture medium increases Ca^2+^ influx in more cells, which in turn increases somatic embryogenesis.

It is interesting to note that increasing Ca^2+^ levels by adding both CaCl_2_ and ionophore A23187 had positive effects only when applied during the first days of culture, when cell clumps are converting into proembryogenic masses and early embryos. The application of increased Ca^2+^ levels for longer times or continuously did not have any significant effect in embryo yield, which implies that the observed positive effect of increased Ca^2+^ levels during the first stages is compensated by a detrimental effect during later stages of embryo development in order to produce no significant final changes. In turn, a reduction of intracellular Ca^2+^ by chelation with EGTA produced similarly detrimental effects when applied during 3 days, 7 days or continuously, which indicates that the detrimental effect seems to be exerted principally during the first stage of embryo induction. Thus, increasing the intracellular Ca^2+^ levels during the inductive stage, when the first embryogenic cells differentiate from cell clumps/proembryogenic masses, increases the percentage of cells effectively induced to embryogenesis, and decreasing the levels has the opposite effect. However, once embryos are induced, during the embryo development stages, any alteration of Ca^2+^ homeostasis would have detrimental effects on embryo production. This is indicating that Ca^2+^ has a key role during the induction of somatic embryogenesis from carrot cells, and that Ca^2+^ homeostasis at this stage is plastic and can be altered to modulate the induction rate, but only during this stage. Once embryos are induced, Ca^2+^ homeostasis must be strictly maintained in order to allow for an efficient embryo maturation, since fully functional, differentiated embryos are able to autonomously regulate their Ca^2+^ levels in their different organs and any external interference may be detrimental.

## Data availability statement

The original contributions presented in the study are included in the article/**Supplementary Material**. Further inquiries can be directed to the corresponding authors.

## Author contributions

Conceptualization: JMSS. Funding Acquisition: JMSS, RM. Investigation: AC-S, PA, RM. Methodology: AC-S, PA, RM. Data curation: AC-S, PA, RM. Formal analysis: AC-S, RM. Project Administration: JMSS. Supervision: JMSS, RM. Writing – Original Draft Preparation: AC-S, JMSS, RM. Writing – Review & Editing: AC-S, JMSS, RM. All authors contributed to the article and approved the submitted version.
