# Supplementary material for: Calcium dynamics and modulation in carrot somatic embryogenesis
Source: Front Plant Sci. 2023 Mar 31;14:1150198. doi: 10.3389/fpls.2023.1150198 (PMC10102378; doi:10.3389/fpls.2023.1150198)
Supplement: Supplementary file 1 [file DataSheet_1.pdf]

Supplementary Material

Calcium dynamics and modulation in carrot somatic embryogenesis

Antonio Calabuig-Serna, Ricardo Mir*, Paloma Arjona, Jose María Seguí-Simarro*

*** Correspondence:** Corresponding authors: seguisim@btc.upv.es; rimimo@upv.es.

1 Supplementary Figures

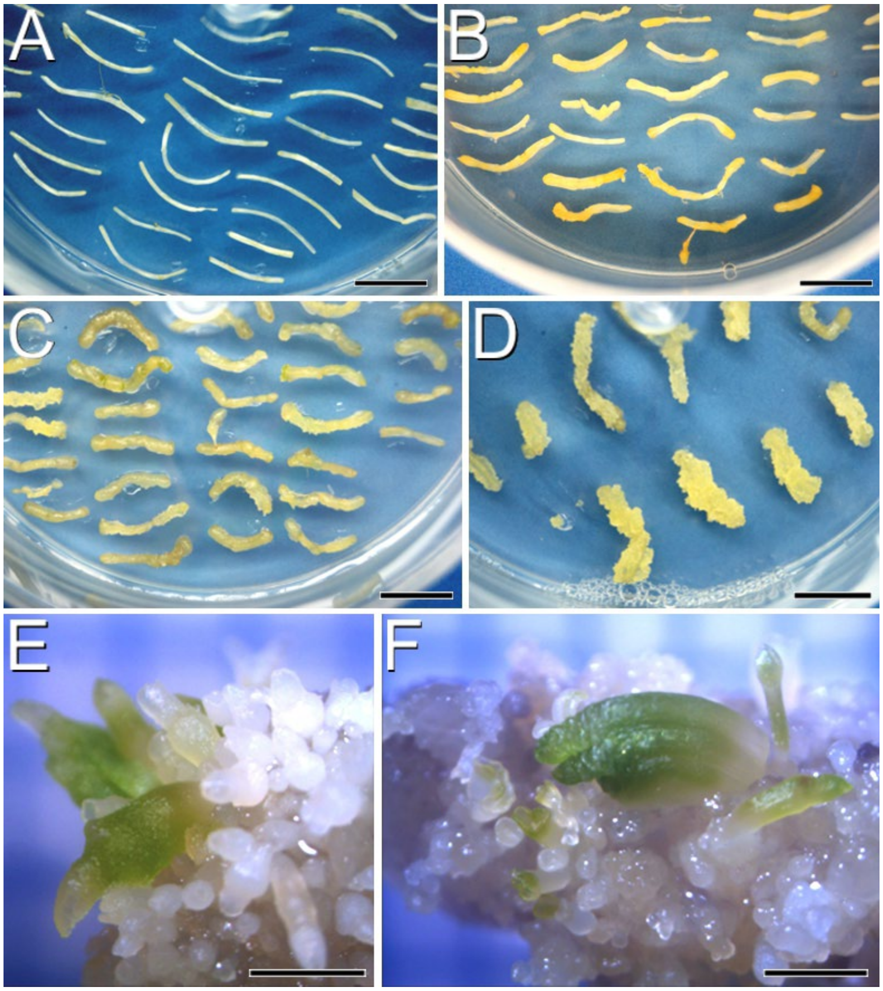

Suppl. Fig. S1. **Carrot transformation and regeneration using protocol D. A**: Fresh hypocotyl explants just transferred to MI medium. **B**: Hypocotyl explants after 15 days in MI medium. Note their color change from whitish to greenish and their swelling as a consequence of callus growth. **C**: Explants after 15 days in MII medium, swollen and covered by callus tissue. **D**: Explants transformed with the PM-YC3.6-LTI6b construct after one month in MII medium, where embryogenic structures developed on the surface of calli. **E**: Emerging embryogenic structures. **F**: Embryos from explants transformed with the PM-YC3.6-LTI6b construct at different developmental stages. Bars: A-D: 1 cm; E, F: 1 mm

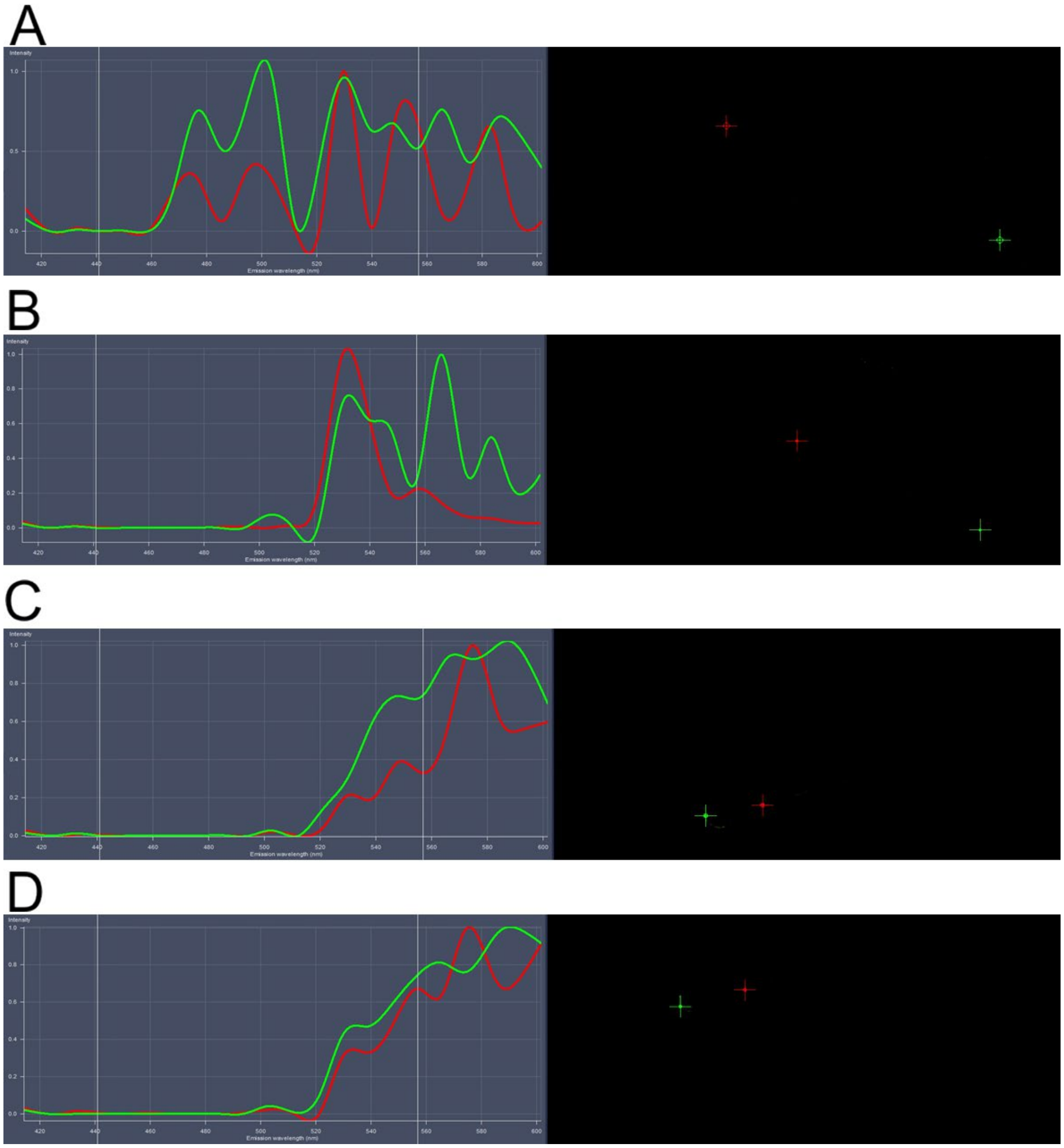

Suppl. Fig. S2. Emission spectrum and confocal images of *D. carota* cell cultures. **A**: Signal of a transformed cell irradiated at 440 nm, the excitation wavelength of CFP. Transformed cell cultures presented a double emission peak at 470 and 500 nm, corresponding to the emission spectrum of CFP, and another peak at 530 nm, corresponding to the emission of YFP resulting from FRET. **B:** Signal of a transformed cell irradiated at 514 nm, the excitation wavelength of YFP. Transformed cultures showed an emission peak at 530 nm, corresponding to the maximum emission peak of YFP. The red and green crosses in the right column images indicate two different plasma membrane regions from two different cells analyzed. When transformed cells are excited at 440 nm (A), three specific peaks are recorded: two at 475 and 500 nm, corresponding to the emission of CFP, and a third at 530 nm corresponding to the emission of the YFP excited by the CFP emission (resonance energy transfer). When transformed cells are excited at 514 nm (B), a specific peak at 530 nm, corresponding to the emission of YFP, is recorded. **C, D**: Signal of non-transformed (control) cells irradiated at 440 nm, the excitation wavelength of CFP (C), and at 514 nm, the excitation wavelength of YFP (D**)**. Note that only unspecific autofluorescence is detected, and no sharp emission corresponding to the specific peaks of CFP and YFP emission described above are observed in these cells.

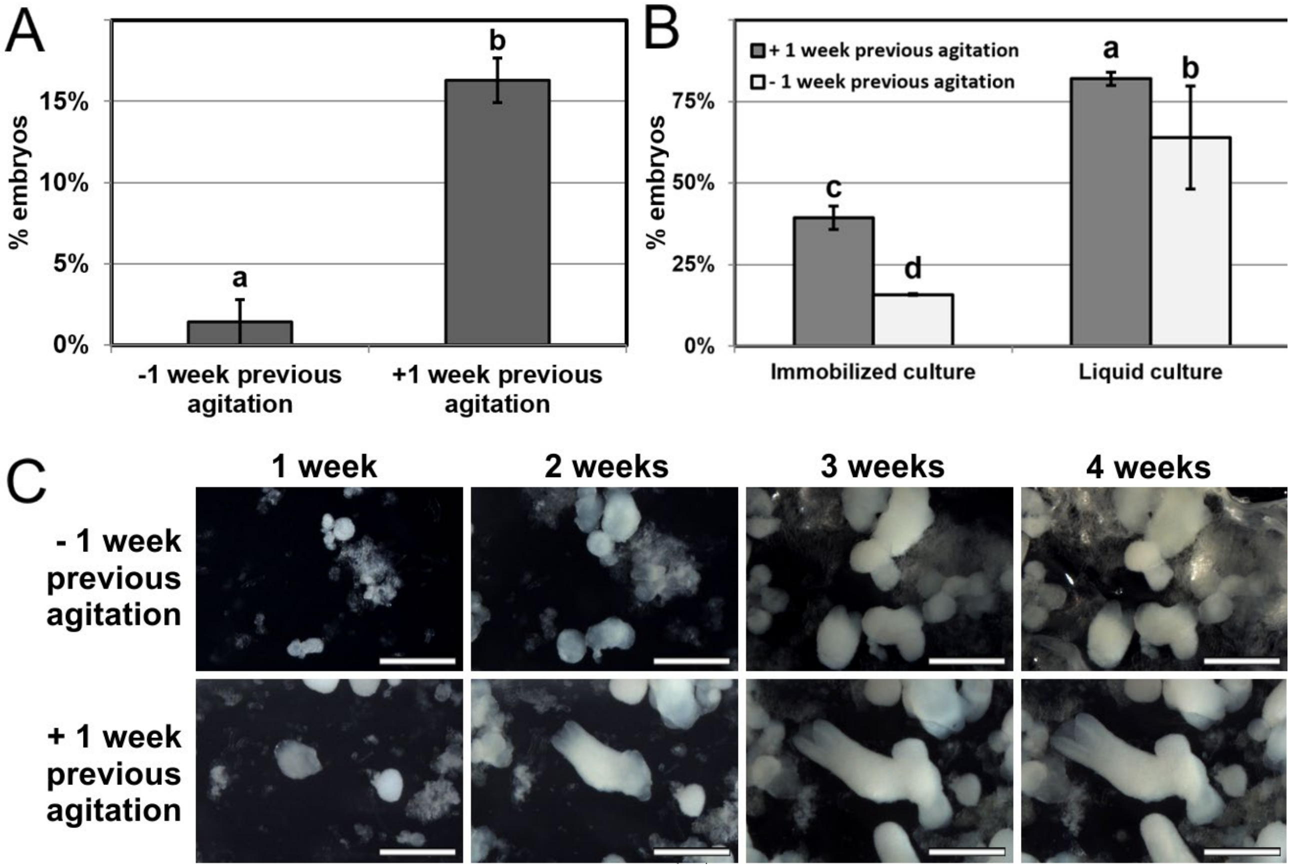

Suppl. Fig. S3. Development of carrot somatic embryos with different culture protocols. A: Comparison of the efficiency of somatic embryogenesis, expressed in percentage of embryos produced (% embryos), either including or excluding a step of one-week culture in liquid medium with agitation prior to transference to the final liquid culture medium. **B**: Comparison of the efficiency of somatic embryogenesis between immobilized cultures and conventional culture in liquid medium, either including or excluding a step of one-week culture in liquid medium with agitation prior to transference to the final immobilized or conventional liquid culture medium. **C**: Time-lapse images taken at one-week intervals of the progression of immobilized cultures either including or excluding a step of one-week culture in liquid medium with agitation prior to transference to the final immobilized medium. Bars: 500 µm.

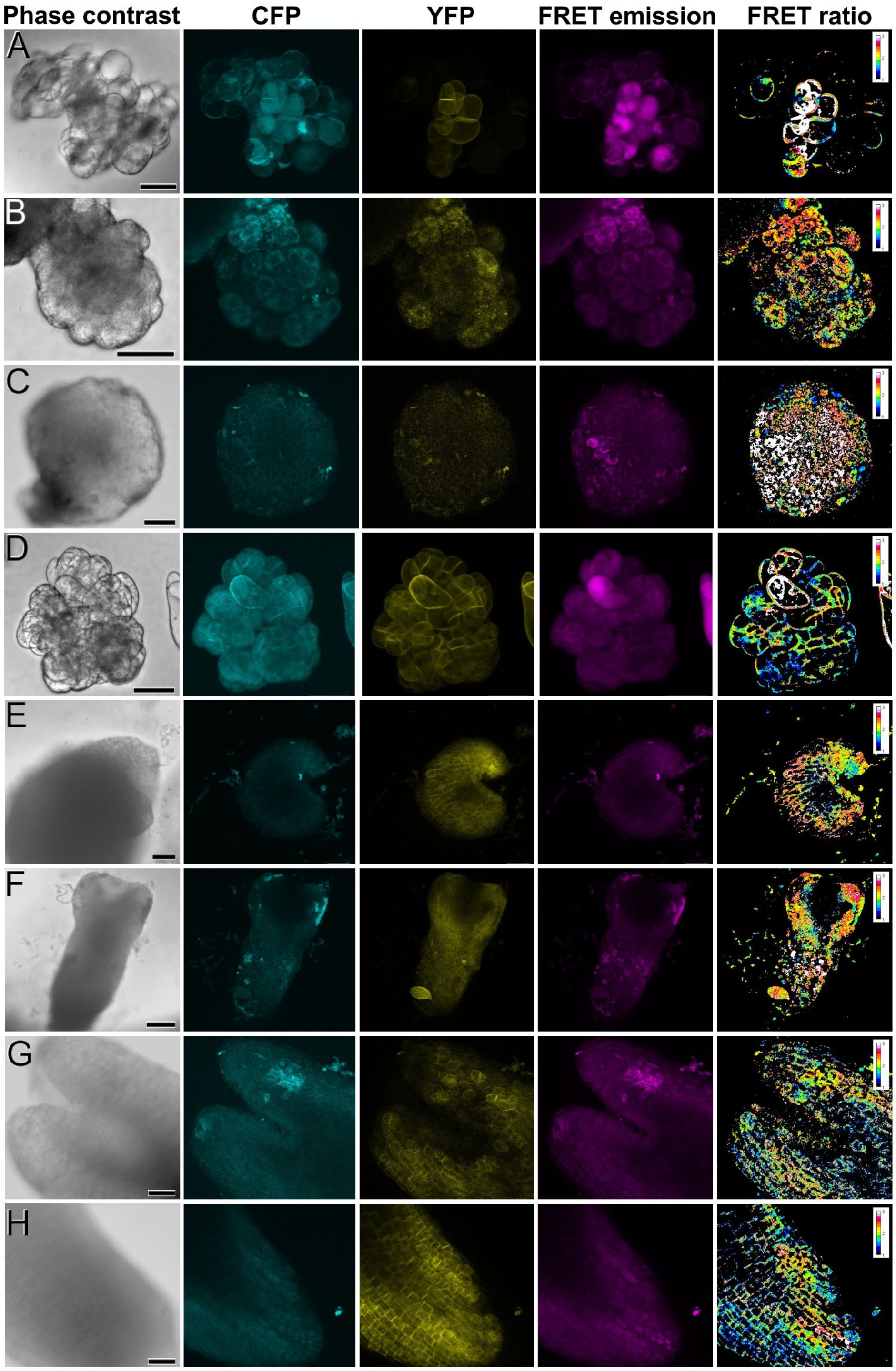

Suppl. Fig. S4. FRET imaging of Ca^{+2} levels during carrot somatic embryogenesis in *cameleon* lines. Each set of images show the same stages shown in Fig. 3 imaged by phase contrast, CFP, YFP, FRET emission fluorescence and FRET (YFP/CFP emissions) ratio. The LUT bar displays the false coloration of FRET ratios. **A**: One-week-old cell clumps. **B:** Cell clump transforming into an embryogenic mass. **C, D**: Two-weeks-old compact, globular embryo (C) and callus mass (D). **E**: Three-weeks-old heart-shaped embryo. **F**: Torpedo embryo. **G, H**: Shoot apical (G) and root (H) regions of a cotyledonary embryo. Bars: A-E, G, H: 40 μm; F: 100 μm.
